# Absorption and Elimination of Oat Avenanthramides in Humans after Acute Consumption of Oat Cookies

**DOI:** 10.1155/2017/2056705

**Published:** 2017-12-21

**Authors:** Tianou Zhang, Jing Shao, Yike Gao, Chi Chen, Dan Yao, Yi Fang Chu, Jodee Johnson, Chounghun Kang, Dongwook Yeo, Li Li Ji

**Affiliations:** ^1^Laboratory of Physiological Hygiene and Exercise Science, School of Kinesiology, University of Minnesota, Minneapolis, MN 55455, USA; ^2^Department of Food Science and Nutrition, College of Food, Agriculture and Animal Science, University of Minnesota, St. Paul, MN 55108, USA; ^3^Quaker Oats Center of Excellence, PepsiCo Nutrition Sciences, Barrington, IL 60010, USA; ^4^Department of Physical Education, Inha University, Incheon, Republic of Korea

## Abstract

**Background:**

Avenanthramides (AVA) are a group of diphenolic acids found only in oats that have anti-inflammatory and antioxidant effects. Absorption of AVAs in humans after oral consumption of natural oat flour is unknown.

**Objective:**

To examine the appearance of AVAs in plasma after oral ingestion of oat cookies and estimate key pharmacokinetic parameters.

**Methods:**

Male and female nonobese participants (*n* = 16) consumed three cookies made with oat flour containing high (229.6 mg/kg, H-AVA) or low (32.7 mg/kg, L-AVA) amounts of AVAs, including AVA-A, AVA-B, and AVA-C. Blood samples were collected at 0, 0.5, 1, 2, 3, 5, and 10 h after ingestion. Plasma total (conjugated and free) AVA concentrations were quantified using UPLC-MS, and pharmacokinetic parameters for each AVA were estimated.

**Results:**

AVAs reached peak concentrations in plasma between 2 and 3 h for the H-AVA group and between 1 and 2 h for the L-AVA group. Maximal plasma concentrations for AVAs were higher in the H-AVA than in the L-AVA group. AVA-B demonstrated a longer half-life and slower elimination rate than AVA-A and AVA-C.

**Conclusions:**

AVAs found naturally in oats are absorbed in the plasma after oral administration in humans. AVA-B has the slowest elimination rate and the longest half-life compared to AVA-A and AVA-C, while AVA-C demonstrated the lowest plasma concentrations. This study is registered with ClinicalTrials.gov identifier NCT02415374.

## 1. Introduction

Avenanthramides (AVAs) are a group of diphenolic acids found only in oats (*Avena sativa*) [1]. Of all the AVAs identified, AVA-A (2p), AVA-B (2f), and AVA-C (2c) are the most abundant and differ only by a single moiety on the hydroxycinnamic acid ring. Early research indicates that oat bran contains a high concentration of antioxidants [2], and increased oat consumption is associated with decreased serum lipid and cholesterol levels [3] and low-density lipoprotein oxidation [4]. Additional in vitro studies show that AVAs have the anti-inflammatory and antiatherogenic effects of decreasing monocyte adhesion to aortic endothelial cells and decreased expression of proinflammatory (e.g., tumor necrosis factor- (TNF-) *α*, interleukin- (IL-) 1*α*, IL-6) markers and chemotactins (e.g., intercellular adhesion molecule-1, vascular cell adhesion molecule-1, monocyte chemotactic protein-1, and c-elastin) [5]. These effects are derived from decreased nuclear factor- (NF-) *κ*B activity [6, 7]. Ji et al. [8] first showed that rats fed with an AVA 2c-supplemented diet for 50 days display lower reactive oxygen species (ROS) generation and lipid peroxidation in skeletal muscle and higher levels of superoxide dismutase (SOD) and glutathione peroxidase activity in the muscle, heart, liver, and kidney. Also, Koenig et al. report that 8 weeks of AVA supplementation among young and postmenopausal women abolishes plasma TNF-*α* and C-reactive protein responses to downhill running-induced inflammation. Compared with women in the low AVA group, women in the high AVA group showed a suppressed neutrophil respiratory burst 0 and 24 h after downhill running and lower IL-6 and monocyte NF-*κ*B activation [9, 10]. These studies show that AVAs may have potential effect to prevent chronic diseases and sports injuries.

AVA bioavailability has been demonstrated in animals and humans. Chen et al. [11] reported that serum levels of AVA 2p, 2f, and 2c reach a peak 2 h after humans consume an AVA-enriched mixture (AEM) and then gradually return to resting levels after 10 h. We previously found that rats orally gavaged with a mixture of synthetic AVAs show significant quantities of all three AVA fractions in the plasma, heart, liver, and skeletal muscle during a 48-hour period [12]. Using glucuronidase-sulfatase to cleave AVA conjugate bonds, we confirmed that most AVA was in a conjugated form. Although previous studies report that AVAs are bioavailable and detectable in humans [11, 13], these studies utilized an AEM or specially treated oats, which might contain much higher amounts of AVAs than regular oats.

Here, we examined the metabolic fate of orally ingested oat AVAs by measuring plasma concentrations of three major fractions of AVAs, AVA-A, AVA-B, and AVA-C. Our results provide the first evidence of AVA absorption in humans after acute consumption of oat cookies made with natural oat flour.

## 2. Methods

### 2.1. Participants and Study Design

Male and female nonobese participants (body mass index (BMI) greater than 18 kg/m^2^ and less than 28 kg/m^2^, age: 20–45 years, *n* = 16) were recruited from the Minneapolis-St. Paul community. All participants signed informed consent forms and were willing to avoid oat consumption at least 24 hours prior to and throughout the testing period and to consume a low-flavonoid diet 1 week prior to the test. This study was approved by the Institutional Review Board of the University of Minnesota and was registered at ClinicalTrials.gov (NCT02415374). Foods rich in flavonoids were defined as having berries, apples, pears, citrus fruits, fruit juices, onions, chocolate, wine, coffee, tea, beans, nuts, soy products, and most spices. Exclusion criteria included the presence of gastrointestinal conditions that interfere with absorption; clinically significant endocrine, cardiovascular, pulmonary, renal, hepatic, pancreatic, biliary, or neurologic disorders; major trauma or surgery within 3 months of the test; cancer within 2 years of the test; allergy to oat products; pregnancy or currently lactating; smoking; drinking more than five alcoholic drinks per week; using nutraceuticals, blood pressure medication, nonsteroidal anti-inflammatory drugs (>800 mg ibuprofen per week), vitamin supplements, anticoagulants, or hypoglycemic drugs; oat product consumption at least 24 hours before the test; and consuming high-flavonoid foods 1 week prior to the test.

### 2.2. Diet

Participants received three cookies made with oat flour containing a high amount of AVA (H-AVA; 7.2 mg/cookie; 21.6 mg total, PepsiCo Inc., USA) or a low amount of AVA (L-AVA; 1.03 mg/cookie; 3.09 mg total, PepsiCo Inc., USA). H-AVA and L-AVA oat flour are compared and selected from 26 natural oat groats incorporating different AVA contents. The concentrations of AVA in the two types of cookies were verified by our laboratory and International Chemistry Testing (Milford, MA). Each cookie was made of 30 g flour, 1 tsp unsweetened applesauce (8 g), 1 tsp noncaloric sweetener (3 g), and 2 tsp water (14 g) and was baked at 212°F (100°C) for 15 min to avoid excessive AVA disintegration.

### 2.3. Study Visits and Blood Collection

After an overnight fast with water allowed, participants reported to the Laboratory of Physiological Hygiene and Exercise Science in the morning. Participants completed a food history questionnaire to ensure that they met the selection criteria; those who consumed vegetables, fruits, or other food containing over 200 mg of polyphenols during the previous week were excluded. Over-the-counter and prescription medication consumed within the past 4 weeks was recorded.

Before cookie consumption, mixed venous blood was drawn from an antecubital vein into an EDTA-coated Vacutainer tube (7 ml) and immediately centrifuged at 500 ×g for 30 min at 20°C to obtain plasma. Participants then consumed three cookies containing either H-AVA or L-AVA in random order with 150 ml water within 10 min. After 0.5, 1, 2, 3, and 5 h, additional blood samples were drawn as described above. The final blood sample was drawn when participants returned to the lab after 10 h. Samples were stored at −80°C until analysis. Participants were retested after a 2-week washout period after consuming three cookies containing the opposite concentration of AVA.

### 2.4. Plasma AVA Extraction

AVA was extracted from plasma according to the procedures described by Chen et al. [4] with modification. Briefly, 50 *μ*l vitamin C-NaH_2_PO_4_ and 50 *μ*l *α*-glucuronidase/sulfatase were added to 500 *μ*l plasma, and the mixture was incubated at 37°C for 45 min. Next, 2000 *μ*l acetonitrile was added to a 5 ml Eppendorf tube and vortexed. After 5 min, samples were centrifuged at 5000 rpm for 30 min. The supernatant was removed, dried by nitrogen, and reconstituted in 100 *μ*l MeOH:H_2_O (1 : 1). Following centrifugation at 14800 rpm for 15 min, 50 *μ*l of the supernatant was transferred into a clean vial for analysis.

### 2.5. UPLC-MS Detection

Concentrations of total AVA in plasma were measured by ultra-performance liquid chromatography quadrupole time-of-flight mass spectrometry (UPLC-Q-TOF MS; Waters XEVO G2-S Q-TOF) using a C18 column (Acquity UPLC BEH C18, 1.7 *μ*m, 2.1 × 50 mm). Gradient elution composing solvent A (0.1% formic acid in H_2_O) and B (0.1% formic acid in acetonitrile) was performed starting with 0.5% solvent B for 0.5 min, gradually increased to 20% solvent B for 3.5 min, and to 95% solvent B for 4 min, and to 100% solvent B for 1 min. Solvent B was decreased from 100% to 0.5% in the last 1 min. The injection volume was 5 *μ*l for each sample. The mass range was measured from 50 to 1000 m/z. Instrument control and data acquisition were performed using Waters MassLynx MS software (version 4.1). The concentrations of AVAs in samples were calculated using an AVA standard curve generated with synthetic standards provided by Dr. Mitchell Wise (USDA Cereal Research Laboratory, Madison, WI). The sensitivity of detection was in the ng/ml range.

### 2.6. Pharmacokinetic Parameters

Maximum plasma concentration (*C*_max_) and time to reach maximal concentration (*T*_max_) of AVAs were calculated and presented as mean ± standard deviation (SD). Half-life (i.e., time taken for concentration to decrease to half the initial value; *T*_1/2_) was calculated from log-transformed plasma concentration-time data and presented as mean ± SD. Area under the curve (AUC) for each participant was calculated using the trapezoidal rule, and mean AUC_0–*t*_ was presented as mean ± SD. Elimination rate constant (K_el_) was calculated using the following equation: *K* = 0.693/*T*_1/2_. *C*_max_, *T*_max_, *T*_1/2_, and AUC_0–*t*_ were calculated with PKSolver, an add-in program for Microsoft Excel [14].

### 2.7. Statistical Analysis

The mean and SD of plasma total (conjugated and free) AVA was calculated for each time point and plotted against time. AUC, *C*_max_, *T*_max_, and *T*_1/2_ for each AVA were compared between H-AVA and L-AVA groups using *t*-tests and among AVA-A, AVA-B, and AVA-C compounds using one-way analysis of variance (ANOVA). *P* < 0.05 was considered statistically significant.

## 3. Results

### 3.1. AVA Content in H-AVA and L-AVA Cookies

Table 1 shows the total AVA content in H-AVA and L-AVA cookies. Participants in the H-AVA group consumed higher amounts of AVA-A, AVA-B, and AVA-C than participants in the L-AVA group.

### 3.2. Participant Characteristics

Table 2 shows the average age (range: 24.9 to 26.3 years) and BMI (range: 21.4 to 23.8 kg/m^2^) of participants, which did not differ between males and females.

### 3.3. TOF-MS Chromatographs of AVAs

**Figure 1:**
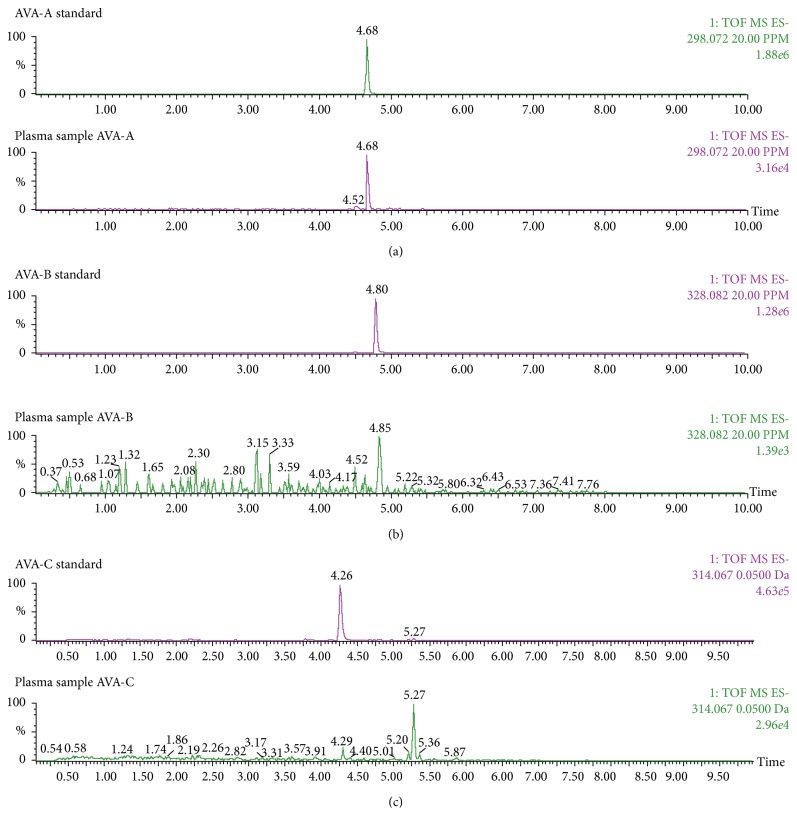
Plasma AVA (bottom) and AVA standard (top) TOF-MS chromatographs. (a) The retention times for AVA-A standard and plasma AVA-A were both 4.68 min. (b) The retention times for AVA-B standard and plasma AVA-B were 4.80 min and 4.85 min, respectively. (c) The retention times for AVA-C standard and plasma AVA-C were 4.26 min and 4.29 min, respectively.

Representative retention times for AVA-A, AVA-B, and AVA-C in standards as measured by Q-TOF-MS were 4.68 min (Figure 1(a)), 4.80 min (Figure 1(b)), and 4.26 min (Figure 1(c)).

### 3.4. Plasma AVA Concentrations

**Figure 2:**
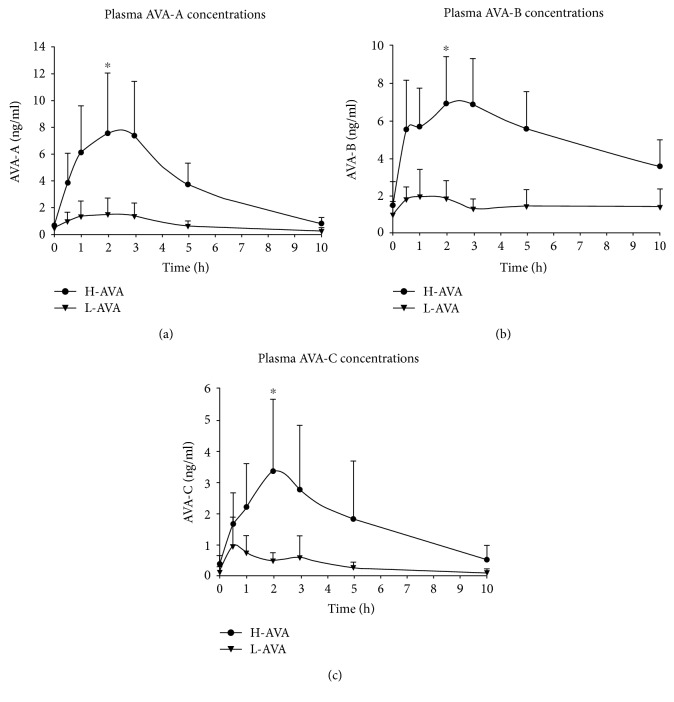
Changes in plasma AVA concentration over time. (a), (b) and (c) represent AVA-A, AVA-B and AVA-C, respectively. Data shown as mean ± SD at each time point. Mean AUC_0–*t*_ was compared between H-AVA and L-AVA for AVA-A, AVA-B, and AVA-C. ^∗^*P* < 0.001, H-AVA versus L-AVA

Participants in the H-AVA group showed higher plasma concentrations of AVA-A, AVA-B, and AVA-C than participants in the L-AVA group. AVA-A concentration reached maximum at 2.50 ± 1.2 h in the H-AVA group and 1.80 ± 1.3 h in the L-AVA group and returned to baseline after approximately 10 h (Figure 2(a)). AVA-B concentrations reached maximum values at 2.04 ± 0.9 h in the H-AVA group and 1.50 ± 1.5 h in the L-AVA group but did not return to baseline levels after approximately 10 h (Figure 2(b)). AVA-C concentrations reached maximum values at 2.29 ± 1.0 h in the H-AVA group and 1.32 ± 1.0 h in the L-AVA group and returned to baseline levels after approximately 10 h (Figure 2(c)).

### 3.5. AVA Pharmacokinetics

Table 3 lists kinetic parameter determined for the three AVA fractions. Maximum plasma concentrations (*C*_max_) for AVA-A, AVA-B, and AVA-C were higher in the H-AVA than in the L-AVA group. Also, AVA-A and AVA-B had higher *C*_max_ than AVA-C in the H-AVA group. AVA-A, AVA-B, and AVA-C were present at peak concentrations in plasma (*T*_max_) between 2 and 3 h for the H-AVA group and between 1 and 2 h for the L-AVA group. AVA-B had longer *T*_1/2_ than AVA-A and AVA-C in the H-AVA group.

Participants in the H-AVA group demonstrated larger mean AUC_0–*t*_ for AVA-A, AVA-B, and AVA-C compared with participants in the L-AVA group (Table 4). AVA-C had the lowest AUC_0–*t*_ among the three AVAs no matter in H-AVA or L-AVA groups.

## 4. Discussion

Oats (*Avena sativa*) are rich in dietary fibers and known for its cardiac protection attributed to the abundant soluble fiber *α*-glucan [15]. Recently, other components in oat with additional health benefits have been further investigated, including vitamin E, phenolic compounds (e.g., AVA), phytic acids, sterols, and flavonoids [16]. AVAs are a group of diphenolic acids unique to oats that exert anti-inflammatory and antiatherogenic effects by decreasing expression of proinflammatory markers (e.g., TNF-*α* and IL-6) [5], possibly due to inhibition of NF-*κ*B pathway [6, 7]. To elucidate absorption and elimination profiles of oat AVAs, we measured plasma concentrations of AVAs in humans after acute ingestion of oat cookies made of natural oat flour.

H-AVA group was found longer *T*_max_ and higher *C*_max_ than L-AVA group in this study. Chen et al. [11] first reported bioavailability of AVAs in humans after consumption of 1 g AEM and found that *C*_max_ was 112.1 ng/ml, 31.6 ng/ml, and 28.1 ng/ml for AVA-A, AVA-B, and AVA-C, respectively. The discrepancies on plasma concentrations were mainly due to the doses of AVA-A, AVA-B, and AVA-C in AEM, which were 9-fold, 4-fold, and 4-fold higher than our H-AVA cookies made of natural oat flour. Other pharmacokinetic properties such as *T*_max_ and *T*_1/2_, however, were similar between the two studies.

H-AVA group exhibited a transient plateau in AVA-B concentration around 30 min to 1 h, which might be related to high absorption of AVA-B after oat flour cookie consumption. Moreover, the absorption profile of AVA-B differed from that of AVA-A and AVA-C, with the highest *C*_max_, longest *T*_1/2_, and shortest *T*_max_. These differences might be related to the different hydroxycinnamic moieties between AVA-A (-H), AVA-B (-OCH_3_), and AVA-C (-OH). That is, as AVA-B is more hydrophobic than AVA-A and AVA-C, this may lead to a slower elimination rate [11]. However, Koenig et al. [12] found that AVA-B showed the fastest elimination rate in rats. This discrepancy between human and rat studies might be due to species differences in phase I and II metabolism [17].

Although participants received a higher dose of AVA-C than AVA-A, plasma concentrations of AVA-C were much lower than those of AVA-A. Likewise, AVA-C showed lower absorption than AVA-A and AVA-B, suggesting its metabolism may be different from the traditional liver phase II pathway. Wang et al. showed that oat AVA-C can be biotransformed and metabolized by mouse and human microbiota into reduction product dihydroavenanthramide- (DH-) 2c, hydrolysis product caffeic acid (CA), and reduction of hydrolysis product DH-CA [18]. The hydrolysis of AVA-C could also explain why plasma concentrations of AVA-C were lower than those of AVA-A and AVA-B. In this study, we also observed that concentrations of plasma caffeic acid (an AVA-C hydrolysis product), but not concentrations of AVA-A and AVA-B hydrolysis products, increased after oat cookie consumption (data not shown), which might explain the relatively low plasma concentration of AVA-C. In addition, the pattern of differences in AUC for AVA-A and AVA-B between H-AVA and L-AVA groups is not consistent with the doses received, which might also be evidence for metabolic variations between different AVAs. Further studies are required to confirm this hypothesis.

We found that L-AVA group had shorter *T*_max_ for AVA-A, AVA-B, and AVA-C compared with H-AVA group, indicating that AVA absorption might be dose-dependent. L-AVA group showed relatively flat time-dependent concentration curves compared with H-AVA group, with plasma AVA concentrations fluctuating around the MS detection limit (~1 ng/ml), indicating that L-AVA group could serve as an appropriate control group compared with H-AVA group. In addition, variations in plasma AVA concentrations among individual participants were relatively large, reaching 50% SD, especially 1–3 h after oat cookie consumption. This variation is similar to that reported in a study on the bioavailability of AVA after acute consumption of “false malted” oat bran, which is high in endogenous AVA [13]. In addition, we also compared the pharmacokinetic parameters between male and female subjects within H-AVA and L-AVA groups (results are not shown) and found some significant differences between female and male subjects, especially in L-AVA group. Female subjects tend to have higher *C*_max_, mean AUC, and shorter *T*_1/2_ than male subjects, indicating female subjects might have higher absorption profile over time and faster absorption rate on AVA than male subjects. However, to clarify these phenomena or mechanisms, further investigations with a more sensitive equipment, more time points, and higher AVA doses are required to compare the dose-response curves.

One limitation in this study was the lack of determination on absolute and relative bioavailability of AVAs. The bioavailability was not calculated because we did not have intravenous AVA doses or other comparable biological products for oral intake. In conclusion, we found that AVAs consumed from cookies made with natural oat flour are absorbed in humans. AVA-B demonstrated the slowest elimination rate and longest *T*_1/2_, perhaps due to its more hydrophobic hydroxycinnamic moiety. Also, AVA-C was estimated to have the lowest relative absorption compared to AVA-A and AVA-B, possibly due to its biotransformation or hydrolysis process. These data demonstrated that AVAs are bioavailable to human, and its previously proved anti-inflammatory and antioxidant effects make it a useful natural phytochemical in sports science and chronic disease prevention. Further investigations are needed to determine the bioavailability of other AVA compounds and the mechanisms contributing to the different absorption and elimination profiles of various AVAs.

## Figures and Tables

**Table 1:** AVA content in H-AVA and L-AVA cookies.

	AVAs	One cookie (55 g)	Per 100 g	Per serving (3 cookies, 165 g)
H-AVA	Total	7.20	13.10	21.61
	A (mg)	1.70	3.09	5.09
	B (mg)	2.89	5.25	8.67
	C (mg)	2.62	4.76	7.85

L-AVA	Total	1.03	1.86	3.08
	A (mg)	0.19	0.35	0.57
	B (mg)	0.32	0.58	0.95
	C (mg)	0.52	0.94	1.55

**Table 2:** Participant characteristics.

	Male (*n* = 8)	Female (*n* = 8)
Age (years)	26.3 ± 3.0	24.9 ± 3.4
BMI (kg/m^2^)	23.8 ± 3.0	21.4 ± 2.2

**Table 3:** Pharmacokinetic properties of AVAs.

	*C*_max_ (ng/ml)		*T*_max_ (h)		*T*_1/2_ (h)	
	H-AVA	L-AVA	H-AVA	L-AVA	H-AVA	L-AVA
AVA-A	8.39 ± 4.2^c^	1.98 ± 1.3^∗^	2.50 ± 1.2	1.80 ± 1.3	2.16 ± 0.6^b^	2.44 ± 0.8
AVA-B	8.44 ± 2.3^c^	2.43 ± 1.1^∗^	2.04 ± 0.9	1.50 ± 1.5	4.23 ± 0.8^ac^	4.60 ± 2.7
AVA-C	4.26 ± 2.0^ab^	1.33 ± 0.7^∗^	2.29 ± 1.0	1.32 ± 1.0	2.71 ± 1.2^b^	2.87 ± 1.4

^∗^ *P* < 0.001 versus H-AVA; ^a^*P* < 0.05 versus AVA-A within H-AVA or L-AVA group; ^b^*P* < 0.05 versus AVA-B within H-AVA or L-AVA group; ^c^*P* < 0.05 versus AVA-C within H-AVA or L-AVA group.

**Table 4:** Plasma absorption of AVAs.

	Mean AUC_0–*t*_ (h × ng/ml)		Mean K_el_ (h^−1^)		Dose (ng × 10^6^)	
	H-AVA	L-AVA	H-AVA	L-AVA	H-AVA	L-AVA
AVA-A	42.56 ± 17.9^bc^	8.50 ±4.4^∗^^b^	0.35 ± 0.1^b^	0.32 ± 0.1^b^	5.09	0.57
AVA-B	53.17 ± 12.1^ac^	6.31±1.6^∗^^ac^	0.17 ± 0.1^ac^	0.20 ± 0.1^ac^	8.67	0.95
AVA-C	19.81 ± 12.7^ab^	3.88±2.0^∗^^b^	0.30 ± 0.1^b^	0.30 ± 0.2^b^	7.85	1.55

^∗^ *P* < 0.001 versus H-AVA; ^a^*P* < 0.05 versus AVA-A within H-AVA or L-AVA group; ^b^*P* < 0.05 versus AVA-B within H-AVA or L-AVA group; ^c^*P* < 0.05 versus AVA-C within H-AVA or L-AVA group.
